# Apoptotic Cell-Derived Extracellular Vesicles: More Than Just Debris

**DOI:** 10.3389/fimmu.2018.01486

**Published:** 2018-06-28

**Authors:** Sarah Caruso, Ivan K. H. Poon

**Affiliations:** Department of Biochemistry and Genetics, La Trobe Institute for Molecular Science, La Trobe University, Melbourne, VIC, Australia

**Keywords:** apoptotic bodies, apoptotic microvesicles, extracellular vesicles, immunomodulation, apoptosis

## Abstract

The many functions of extracellular vesicles (EVs) like exosomes and microvesicles released from healthy cells have been well characterized, particularly in relation to their roles in immune modulation. Apoptotic bodies, a major class of EV released as a product of apoptotic cell disassembly, and other types of EVs released from dying cells are also becoming recognized as key players in this emerging field. There is now increasing evidence to suggest that EVs produced during apoptosis have important immune regulatory roles, a concept relevant across different disease settings including autoimmunity, cancer, and infection. Therefore, this review focuses on how the formation of EVs during apoptosis could be a key mechanism of immune modulation by dying cells.

## Immune Regulation by Extracellular Vesicles (EVs)

**Figure 1:**
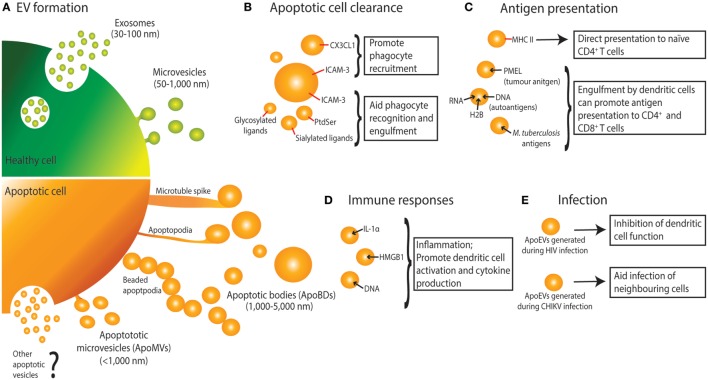
Extracellular vesicle (EV) formation and immune functions of apoptotic cell-derived EVs (ApoEVs). **(A)** Healthy cells form two main types of EV, namely exosomes that are release *via* exocytosis of multivesicular bodies, and microvesicles that are shed from the plasma membrane. During apoptosis, dying cells can also release ApoEVs. Apoptotic cells can undergo morphological changes including membrane blebbing, thin membrane protrusion formation (microtubule spikes, apoptopodia, and beaded-apoptopodia), and generation of distinct apoptotic bodies (ApoBDs). Apoptotic cells can also release EVs that are similar in size as microvesicles (ApoMVs), however, it is unclear if ApoMVs are generated *via* the same mechanism as microvesicles from healthy cells. Whether apoptotic cells can generate vesicles that are similar to exosomes is undetermined. **(B)** ApoEVs can harbor “find-me” signals (e.g., CX3CL1 and ICAM-3) to attract phagocytic cells, as well as “eat-me” signals [e.g., ICAM-3, phosphatidylserine (PtdSer), and sialylated and glycosylated ligands] to promote uptake by phagocytes. **(C)** ApoEVs have MHC II molecules on their surface, which is essential for direct antigen presentation to naïve CD4^+^ T cells and activation of immunological memory. ApoEVs can also carry antigen to professional antigen-presenting cells (e.g., dendritic cells). Antigens carried by ApoEVs include autoantigens, tumor antigens, and microbial antigens. **(D)** ApoEVs can promote inflammation by transporting proinflammatory cytokines such as IL-1α and damage-associated molecular patterns including DNA and HMGB1. **(E)** ApoEVs can aid HIV infection by inhibiting dendritic cell activation. Chikungunya virus (CHIKV) can hijack ApoEVs to propagate infection to neighboring cells.

There are three main types of EVs formed by a cell, namely exosomes, microvesicles, and apoptotic bodies (ApoBDs). These three types of EVs vary in size, content, and mechanism of formation (Figure 1) (1). To date, exosomes and microvesicles generated from healthy cells are more extensively characterized and the formation of these EVs is key in mediating intercellular communication and immune regulation. Exosomes and microvesicles have been shown to play an important role in processes including antigen presentation, immune suppression, antitumor immunity, and autoimmunity. This has been the subject of many reviews, highlighting how EVs modulate immune responses by a myriad of mechanisms (2–5). Of particular interest is how the contents of exosomes and microvesicles enable them to regulate immune cell functions. Notably, these EVs can exhibit immune activating or immune suppressing properties depending on the specific circumstances. For example, exosomes have been shown to either activate or dampen the overall cytokine response through regulation of gene expression in monocytes and release of soluble cytokine receptors, respectively (6, 7). Exosomes derived from dendritic cells, B lymphocytes, and tumor cells have also been shown to regulate immunological memory through the surface expression of antigen-presenting MHC I and MHC II molecules, and subsequently eliciting T cell activation and maturation (8–12). Exosomes can also play a role in cross-presentation pathways and have been shown to promote dendritic cell activation and maturation (12). Furthermore, microvesicles can modulate immune responses by transporting cytokines such as IL-1β (13) and proinflammatory microRNAs (14).

Both exosomes and microvesicles are generally described as EVs released from healthy cells, however, dying cells can also release a variety of EVs, broadly known as apoptotic cell-derived EVs (ApoEVs) (Figure 1) (1, 15, 16). Subtypes of ApoEVs include large membrane-bound vesicles like ApoBDs (15, 17) or smaller apoptotic microvesicles (ApoMVs) (18, 19), both of which are described in detail below. While it has been well established that EVs can exhibit immunomodulatory effects, most studies have focused on EVs released from healthy cells, with EVs released from dying cells largely understudied. Nevertheless, a number of studies have suggested that ApoEVs have similar functional importance as EVs released from healthy cells. ApoEV formation has two key proposed functions: (a) aiding apoptotic cell clearance and (b) means of intercellular communication, both of which have implications in immune regulation. Many cells in the body are constantly undergoing apoptosis, and while a large portion of these are healthy cells undergoing normal turnover, apoptosis also occurs in many immunological and disease settings including inflammation, infection, autoimmunity, and cancer (20–23). Here, we discuss how ApoEVs may act as an immunomodulatory mechanism for apoptotic cells.

## Generation of EVs During Apoptosis

As first described by Kerr et al., during apoptosis a cell undergoes a series of morphological changes resulting in the dismantling of the dying cell (17). Recently, disassembly of the apoptotic cell is categorized into three distinct morphological steps, namely apoptotic membrane blebbing, thin membrane protrusion formation, and ultimately generation of ApoBDs that are generally defined as 1–5 µm in diameter (15, 17) (Figure 1). While less is known about the mechanisms driving the formation of ApoBDs compared to other types of EVs, recent studies suggest that it is a highly regulated process and has been reviewed in detail (15, 16, 24). Besides ApoBDs, cells can also release smaller EVs such as ApoMVs (<1 μm in diameter) during the progression of apoptosis, possibly through membrane budding (18, 19, 25, 26). However, molecular regulators of ApoMVs formation are not well defined.

It is important to note that in the literature there are striking discrepancies in the characterization and isolation of ApoEVs (27, 28). Aside from size, currently there are no well-defined criteria to distinguish ApoBDs from other ApoEVs, in particular ApoMVs. Although proteomic studies comparing these ApoEV subtypes have been performed (25, 26), clear standard for the characterization and purification of ApoEV subtypes is lacking (highlighted in Table 1). These discrepancies make it difficult to draw accurate conclusions regarding the functions of ApoEVs and caution should be taken when interpreting data involving ApoEVs. Taking these limitations into consideration, here we use the general term ApoEVs where it is unclear which subtype of ApoEVs is presented in a given study, and ApoBDs and ApoMVs to describe vesicles predominantly >1–5 µm and <1 μm in diameter, respectively.

**Table 1:** Variation in nomenclature and isolation/characterization methods in articles describing the immunomodulatory properties of ApoEVs.

Author and year	Reference	Nomenclature used by the authors	Summary of main findings	Isolation/characterization method	ApoEV subtype (ApoBDs, ApoMVs, or unclear^a^)
Segundo et al. (1999)	(39)	Apoptotic blebs	Cell-depleted supernatant from apoptotic B cells stimulated macrophage chemotaxis. When the supernatant was passed through a 0.1 µm filter this effect was lost, suggesting larger vesicles are responsible for the observed effect	Centrifugation at 300 *g* to remove cells, followed by 100,000 *g* spin to collect vesicles. Purity of cell-depleted supernatant validated by microscopy	Mix of ApoMVs and ApoBDs
Thery et al. (2001)	(25)	ApoMVs	Proteomics analysis of exosomes and apoptotic vesicles was performed and showed differential enrichment of proteins between each vesicle type. Total vesicle number increased in the apoptotic samples	Isolation of ApoEVs by differential centrifugation (300, 1,200, 10,000, and 110,000 *g*). Vesicles were further characterized by flow cytometry and exposure of surface PtdSer monitored	Mix of ApoBDs and ApoMVs
Schaible et al. (2003)	(57)	Apoptotic vesicles	Apoptotic vesicles from tuberculosis-infected macrophages transferred bacterial antigen to dendritic cells. After engulfment of these apoptotic vesicles, dendritic cells could then crossprime CD8^+^ T cells	Isolation of ApoEVs by differential centrifugation (800, 1,800, 25,000, and 100,000 *g*). Size of vesicles used not described	Unclear
Distler et al. (2005)	(43)	Microparticles	Engulfment of ApoEVs by macrophages induced macrophage apoptosis and the release of microparticles	Centrifugation at 1,500 *g* to remove cells, followed by 100,000 *g* spin to collect vesicles. Vesicles further characterized by flow cytometry	Mix of ApoMVs and ApoBDs
Winau et al. (2006)	(58)	Apoptotic vesicles	Vaccination with apoptotic vesicles protected mice against tuberculosis infection	Isolation of ApoEVs by differential centrifugation (800, 1,800, 25,000, and 100,000 *g*). Size of vesicles validated by EM (approximately 500 nm)	Unclear, likely ApoMVs
Schiller et al. (2008)	(53)	ApoBDs	Autoantigens such as H2B and DNA, RNA were distributed into ApoBDs from lymphoblasts, which were subsequently engulfed by monocyte-derived phagocytes. Lymphoblasts showed an increase in vesicle formation during apoptosis	Centrifugation at 300 *g* to remove cells, and the supernatant passed through a 1.2 µm filter, followed by 100,000 *g* spin to collect vesicle. Large ApoBDs may be excluded. Vesicle size determined by EM (approximate 500 nm)	Mix of ApoMVs and some ApoBDs
Truman et al. (2008)	(37)	Apoptotic microparticles	CX3CL1/fractalkine released as vesicle-associated signal from apoptotic B lymphocytes	Cell-free supernatant was used (procedure not described). Vesicles were further characterized by flow cytometry and exposure of surface PtdSer monitored	Unclear
Fransen et al. (2009)	(36)	Apoptotic blebs	Apoptotic blebs were engulfed more efficiently than apoptotic cells by dendritic cells. Only the blebs but not the apoptotic cells induced dendritic cell maturation and IL-6 release	Apoptotic cells were centrifuged at 1,550 *g* (this pellet is likely to contain large ApoBDs). Supernatant were centrifuged at 15,700 *g* to isolate “apoptotic blebs.” No vesicle size validation described	Mix of ApoMVs and ApoBDs
Reich and Pisetsky (2009)	(52)	Microparticles	Microparticles contained DNA and RNA that antibodies could access	Centrifugation at 400 *g* to remove cells, and the supernatant passed through a 1.2 µm filter. Small ApoBDs may be included. No vesicle size validation described	Mix of ApoMVs and some ApoBDs
Berda-Haddad et al. (2011)	(42)	ApoBDs, microparticles	ApoBDs but not microparticles contained IL-1α and induced neutrophil infiltration *in vivo*	Centrifugation at 300 *g* to remove apoptotic cells, followed by 4,500 and 75,000 *g* spin to collect vesicles. Apoptotic supernatant was analyzed by flow cytometry, and different sized beads were used to identify 1–3 µm events (ApoBDs) and 0.5–1 µm events (microparticles)	ApoBDs and ApoMVs
Krejbich-Trotot et al. (2011)	(71)	Apoptotic blebs	Infection of HeLa cells with Chikungunya virus induced apoptosis and infection of neighboring cells. Blocking blebbing and apoptotic bleb formation decreased infection of neighboring cells	Analyzed vesicle function using inhibitors of membrane blebbing (ROCK1 inhibitors and actin polymerization inhibitors). Vesicle size not determined	ApoBDs, possibly ApoMVs
Bilyy et al. (2012)	(50)	Subcellular membranous particle (scMP)	Glycosylated ligands were detected on the surface of scMP, which acted as an “eat-me” signal for macrophages	Procedure for isolating scMP and vesicle size validation not described. scMP population monitored by flow cytometry	Unclear
Farinacci et al. (2012)	(56)	Apoptotic vesicles	Apoptotic vesicles from tuberculosis-infected macrophages activated dendritic cells following engulfment and subsequently primed CD4^+^ and CD8^+^ T cells	Isolation of ApoEVs by differential centrifugation (800, 1,800, 25,000, and 100,000 *g*). Vesicle size determined by EM (40–250 nm)	ApoMVs
Frleta et al. (2012)	(70)	Apoptotic microparticles	HIV infection induced the production of apoptotic microparticles that could suppress the ability of dendritic cells to prime CD8 T cells	Centrifugation at 400 *g* to remove cells, and the supernatant ultracentrifuged at 100,000 *g*. Vesicles were further characterized by flow cytometry and exposure of surface PtdSer monitored. Vesicle size determined by EM (0.1–1 µm)	ApoMVs and possibly some ApoBDs
Schiller et al. (2012)	(19)	Apoptotic cell-derived membrane microparticles (AdMPs)	Apoptotic microparticles stimulated dose-dependent IFN-α production from plasmacytoid dendritic cells, whereas supernatants from viable or necrotic cells had no effects	Centrifugation at 500 *g* to remove cells, and the supernatant passed through a 1.2 µm filter followed by 100,000 *g* spin to collect vesicles. Small ApoBDs may be included. Vesicles further characterized by flow cytometry	Mix of ApoMVs and some ApoBDs
Torr et al. (2012)	(40)	Apoptotic microparticles	ICAM-3 was lost from the surface of apoptotic cells with the formation of ICAM-3-associated apoptotic microparticles. These vesicles promoted macrophage recruitment, while vesicles from ICAM-3 deficient cells were less effective	Centrifugation at 350 *g* to remove apoptotic cells, and the supernatant was used. Vesicle size determined by dynamic light scattering (average 200 nm in diameter, much smaller than expected based on the isolation procedure)	Mix of ApoMVs and ApoBDs
Fehr et al. (2013)	(63)	Apoptotic cell-derived membrane vesicles, apoptotic blebs	Apoptotic blebs increased expression of dendritic cell activation markers, but decreased MHC II on dendritic cells. Apoptotic blebs-treated dendritic cells failed to induce T cell proliferation	Centrifugation at 500 *g* to remove cells, and the supernatant passed through a 1.2 µm filter. Small ApoBDs may be included. Vesicles further characterized by flow cytometry	Mix of ApoMVs and some ApoBDs
Schiller et al. (2013)	(69)	Apoptotic cell-derived membraneous vesicles (ACMVs)	HMGB1 detected in vesicles generated during apoptosis	Centrifugation at 500 *g* to remove cells, and the supernatant passed through a 1.2 µm filter followed by 100,000 *g* spin to collect vesicles. Small ApoBDs may be included. Vesicles further characterized by flow cytometry	Mix of ApoMVs and some ApoBDs
Eguchi et al. (2015)	(41)	Microparticles	Adipocyte microparticles promoted monocyte chemotaxis both *in vitro* and *in vivo*	The supernatant following centrifugation at 2,000 *g* was used. Exposure of surface PtdSer on vesicles monitored by flow cytometry	Mix of ApoMVs and ApoBDs
Niessen et al. (2015)	(44)	AdMPs	Uptake of apoptotic microparticles by macrophages promoted the release of proinflammatory cytokines IL-6, IL-8, and TNFα	Centrifugation at 500 *g* to remove cells, and the supernatant passed through a 1.2 µm filter followed by 100,000 *g* spin. Small ApoBDs may be included. Size of vesicles used not described	Mix of ApoMVs and some ApoBDs
Zirngibl et al. (2015)	(54)	ACMVs	Autoantigen histone H2B was shown to be loaded into apoptotic vesicles in a cytoskeleton-dependent manner	Monitored apoptotic vesicles by microscopy and classified into small (<1 μm), medium (1–3 µm), or large (>3 μm) vesicles	N/A
Black et al. (2016)	(51)	Apoptotic vesicles	CD169 (macrophage adhesin molecule) on apoptotic vesicles suppressed dendritic cell-mediated cytotoxic T cell response	Isolation of ApoEVs by differential centrifugation (25,000 *g* pellet). Sucrose gradient was used to separate ApoEVs from non-apoptotic material (only used fractions with β-actin). Vesicle size determined by CryoEM (35–814 nm)	ApoMVs
Muhsin-Sharafaldine et al. (2016)	(66)	Apoptotic vesicles, MVs	Apoptotic vesicles were able to activate naïve T cells and stimulate immunological memory *via* vesicle-associated MHC/antigen complex	Centrifugation at 450 and 3,200 *g* to remove cells, followed by a 25,000 *g* spin. Sucrose gradient was used to separate ApoEVs, microparticles, and exosomes. Vesicle size determined by CryoEM and dynamic light scattering (103–816 nm)	ApoMVs
Sisirak et al. (2016)	(64)	Apoptotic microparticles	DNA contained in apoptotic microparticles was shown to be antigenic when not digested by DNase1L3 and could contribute to an SLE-like condition	Centrifugation at 1,500 rpm to remove cells, followed by 22,000 *g* spin to collect vesicles from apoptotic cells generated *in vitro*. Centrifugation at 2,000 *g* to remove cells, followed by 22,000 *g* spin to collect vesicles from plasma. Vesicles further characterized by flow cytometry and exposure of surface PtdSer monitored	Mix of ApoBDs and ApoMVs
Ainola et al. (2017)	(18)	ApoBDs, apoptotic particles, apoptotic microparticles	Noted an increase in vesicles formation (ApoBDs and microparticles) when HeLa cells were exposed to apoptotic stimuli. These vesicles mediated autoantigen transfer to plasmacytoid dendritic cells, resulting in proinflammatory cytokine production. Highlighted differences between subtypes of vesicles generated during apoptosis	Isolation of ApoEVs by differential centrifugation at 357 *g* (cell pellet), 1,400 *g* (ApoBDs), 16,000 *g* (ApoBDs and microvesicles), and 100,000 g (microvesicles). ApoEVs were further characterized by flow cytometry, ApoBDs (1–4 µm), and ApoMVs (0.1–1 µm), based on sizing beads. Authors commented on size overlap and how complete separation is not possible	ApoBDs, ApoMVs, mix of ApoBDs and ApoMVs
Tucher et al. (2018)	(26)	Apoptotic cell-released large EVs and small EVs	Noted an increase in large EVs (200–1,000 nm) being released from T cells following the induction of apoptosis. Performed proteomic analysis on different EV subsets and identified several proteins that may be specific to T cell ApoEVs	Centrifugation at 300 *g* to remove cells, supernatant passed through a 1.2 µm filter. Further centrifugation at 10,000 *g* to pellet large EVs followed by 100,000 *g* to pellet small EVs. Vesicle size distribution determined by nanoparticle tracking analysis	Mix of ApoMVs and some ApoBDs

*^a^Where there was no indication from the purification procedure if the ApoEVs used were ApoMVs (50–1,000 nm) or ApoBDs (1,000–5,000 nm)*.

*ApoMVs, apoptotic microvesicles; ApoBDs, apoptotic bodies; ApoEVs, apoptotic cell-derived EVs; PtdSer, phosphatidylserine; EVs, extracellular vesicles; SLE, systemic lupus erythematous*.

## ApoEVs Aid Removal of Dying Cells

It has been well established that apoptotic cells coordinate a number of intercellular signals to aid in their detection and removal, and these signals are critical to ensure the immunologically silent characteristic of apoptosis (22, 29). Defective apoptotic cell clearance has been identified as a key contributing factor to autoimmune disease, whereby cells that are unable to be cleared efficiently eventually undergo secondary necrosis and release potentially damaging proinflammatory contents and autoantigens (30–32). There has been mounting evidence suggesting that the release of ApoEVs during apoptosis can promote clearance of apoptotic material, with the mechanism underpinning this process discussed below (33–36).

### “Find-Me” Signals in Association With ApoEVs

For efficient apoptotic cell clearance, the recruitment of phagocytic cells toward the site of cell death is essential. To this end, apoptotic cells can release molecular factors known as “find-me” signals to attract phagocytes. Traditional “find-me” signals include the release soluble factors such as ATP, UTP, CX3CL1/fractalkine, and lysophosphatidylcholine (21, 22, 37, 38). However, there is also evidence of ApoEV-associated “find-me” signals being released from apoptotic cells.

While few studies elucidated the specific molecules involved in ApoEV-mediated recruitment of phagocytes, they have demonstrated ApoEVs to exhibit chemoattractive properties (39–41). Nevertheless, the “find-me” signal CX3CL1/fractalkine was found to be released from apoptotic B lymphocytes in association with ApoMVs (37) and the chemoattractive molecule ICAM-3 was associated with ApoEVs generated from apoptotic lymphoma cells (40). It is interesting to note that ApoEVs appear to have preferential recruitment of macrophages but not neutrophils (41). Such selective recruitment of different phagocytes by ApoEVs may be related to the subtype of ApoEVs being released by the apoptotic cell, in which one study comparing endothelial cell-derived ApoEVs of different size showed that only larger ApoEVs (1–3 µm in diameter, corresponding to ApoBDs) promoted neutrophil migration, whereas smaller EVs (<1 μm, corresponding to ApoMVs) could not (42). It is worth noting that this phenomenon has been observed *in vitro* as well as *in vivo*, where intraperitoneal administration of ApoBDs in a mouse model stimulated neutrophil infiltration (42). Thus, different subtype of ApoEVs may have distinct functions in apoptotic cell clearance.

### ApoEV Formation Promotes Engulfment

Besides attracting phagocytes, formation of ApoEVs, in particular cell fragmentation into ApoBDs has been suggested to enhance removal of apoptotic material, an effect probably attributed to the size of ApoBDs being smaller bite-size pieces that can easily be engulfed by phagocytes. Supporting this concept, it has been shown that dendritic cells can more readily engulf smaller ApoBDs than whole apoptotic cells (16, 36). Furthermore, cells undergoing apoptotic cell disassembly and therefore producing ApoEVs are more efficiently engulfed by macrophages (33, 43, 44).

It should be noted that exposure of “eat-me” signals, such as phosphatidylserine (PtdSer), on apoptotic cells label them for clearance by phagocytes (22, 45). Likewise, ApoEVs can also expose “eat-me” signals like PtdSer on their surface and be recognized by macrophages for removal *via* phagocytic receptors such as CD36 (43, 46–49). Interestingly, ApoEVs can also expose ICAM-3, and specific sialylated and glycosylated ligands on its surface to trigger recognition and engulfment by macrophages (40, 50, 51).

## ApoEVs As Key Regulators of Antigen Presentation

An important immunomodulatory property of EVs is their ability to aid antigen presentation, a fundamental process for adaptive immunity. As mentioned above, EVs like exosomes have been shown to mediate antigen presentation *via* direct and cross-presentation mechanisms (8–12). Similarly, ApoEVs can also regulate antigen presentation *via* these mechanisms in a number of disease settings including autoimmunity (18, 52–54), antimicrobial immune responses (55–58), and organ/transplant rejection (59). Direct antigen presentation involves the vesicle carrying surface MHC molecules in complex with antigenic peptide to directly interact with naïve T cells (60). ApoEVs generated from dendritic cells and B16-F1 melanoma cells carried MHC II molecules suggesting the potential of ApoEVs to activate CD4^+^ T cells (61). Alternatively, cross-presentation relies on the vesicle transporting the antigen to professional antigen-presenting cells, in particular dendritic cells, for antigen processing and presentation to CD8^+^ T cells (62). In one study, ApoEVs generated from *Mycobacterium tuberculosis*-infected mouse macrophages were found to transfer bacterial-derived antigens to dendritic cells, and subsequently activate naïve CD8^+^ T cells (57). Furthermore, engulfment of ApoEVs by dendritic cells has also been shown to modulate their antigen-presenting capabilities. ApoEVs generated from lymphoblasts were found to suppress immune responses by downregulating MHC II molecules on dendritic cells (63).

While the mechanisms underpinning the ability of ApoEVs to modulate antigen presentation are diverse, it is clear that ApoEVs can contribute to the development of autoimmunity, and establishment of antitumor and antimicrobial immunity by regulating the antigen presentation process, as discussed further in detail below.

### ApoEVs as Mediators of Autoimmunity

As discussed above, impaired clearance of dying cells is a major factor contributing to the development of autoantibodies in autoimmune conditions (30–32). Although the formation of ApoEVs has been shown to promote apoptotic cell clearance (36, 40, 43, 51) and thus limiting the release on intracellular antigenic and proinflammatory contents, ApoEVs formation has also been proposed as a mechanism of facilitating the transport of autoantigens to antigen-presenting cells and drive autoimmunity. In particular, ApoEVs have been implicated in the development of systemic lupus erythematous (SLE), whereby autoantigens such as histone H2B can be translocated into ApoEVs during the early stages of apoptosis in HeLa cells *via* a microtubule driven mechanism (54). Lymphoblast-derived ApoEVs containing histone were also more readily engulfed by monocyte-derived phagocytes (53). Furthermore, ApoEV-associated autoantigens like DNA can bind directly to antinuclear antibodies (52, 64), a common feature of autoimmune conditions (65). In addition to autoantigens associated with SLE, Sjögren’s syndrome nuclear autoantigens, such as hy1-RNA, are also detectable in both ApoMVs and ApoBDs generated from epithelial cells and can be transferred to dendritic cells *via* these ApoEVs (18).

### Promoting Antitumor Immunity Through ApoEVs

With most cancer treatments focusing on inducing apoptosis in tumor cells, it becomes important to consider how the release of ApoEVs from dying tumor cells will impact the immune response toward the tumor. Recently, it has been shown that ApoMVs derived from tumorigenic apoptotic melanoma cells can promote antitumor immunity, in which mice immunized with ApoMVs generated from B16-F1 cells following doxorubicin treatment were protected against subsequent tumor challenges (61). Importantly, the tumor antigen PMEL was also found in ApoMVs (66), supporting the concept that ApoMVs can facilitate the transport of tumor antigens to antigen-presenting cells to promote antitumor immunity. It is interesting to note that despite ApoMVs having a relatively lower quantity of the tumor antigen PMEL as compared to other EVs like exosomes, the antitumor protective effect of ApoMV immunization was greater (61), suggesting that ApoMVs may aid antigen presentation *via* a different mechanism as other EVs and were able to promote a more robust antitumor immune response. As discussed earlier, “eat-me” signals such as PtdSer are present on ApoEVs (43, 46). Interestingly, another “eat-me” signal, calreticulin, that are exposed on certain apoptotic tumor cells can play a key role in promoting antitumor immunity through dendritic cells (23, 48, 67, 68). Therefore, it would be of interest to determine whether calreticulin is present on ApoEVs and whether exposure of calreticulin is important for ApoEV-mediated antitumor immunity.

### Establishing Antimicrobial Immunity Through ApoEVs

In addition to the presentation of self-antigens, it is important to note that under conditions where infected cells undergo apoptosis, the resultant ApoEVs may also harbor antigens from the infectious agent. The transfer of microbial-derived antigens *via* ApoEVs to antigen presentation cells like dendritic cells have been shown to provide a protective effect for the host. For example, ApoEVs released from apoptotic macrophages infected with the *M. tuberculosis* can be engulfed by peripheral monocyte-derived and splenic dendritic cells, which could subsequently activate the engulfing dendritic cells to prime naïve CD4^+^ or CD8^+^ T cells (56–58). Significantly, ApoEVs generated from *M. tuberculosis*-infected cells were able to be used to vaccinate naïve animals and provided protection against tuberculosis infection, highlighting the potential use of ApoEVs as vaccines (58). While these studies focus on tuberculosis infection, ApoEVs could play an important role in regulating antimicrobial immunity against other pathogens, however, these remain underexplored.

## ApoEVs Modulate Immune Cell Responses

In addition to antigens, ApoEVs can harbor a variety of biomolecules that could directly modulate immune cells, most commonly *via* vesicle-associated cytokines or damage-associated molecular patterns (DAMPs), which could drive inflammation and dictate the immune cell responses. For example, proinflammatory cytokine IL-1α was detected in ApoBDs but not ApoMVs generated from endothelial cells induced to undergo apoptosis by prothrombic and hypoxic conditions *in vitro* (42). In a mouse model, administration of these endothelial cell-derived ApoBDs into the peritoneal cavity was able to induce production of neutrophil chemokines and promote neutrophil infiltration to drive sterile inflammation (42). Furthermore, an increase in IFN-α production by plasmacytoid dendritic cells in response to DNA in lymphoblast-derived ApoMVs was comparatively more pronounced than DNA isolated from whole cells (19). In this case, vesicle-associated DAMPs were responsible in promoting dendritic cell maturation, with the potential to promote damaging inflammation and possibly autoimmune conditions (19, 53). Besides DNA, other DAMPs such as HMGB1 can also be found in ApoEVs derived from peripheral blood mononuclear cells and T cells (26, 69).

## Hijacking ApoEVs During Viral Infections

The potential protective effects of ApoEVs in infection was discussed earlier, however, ApoEVs have also been implicated in facilitating the spread of infection *via* two different mechanisms. First, ApoEVs generated from infected cells could modulate the immune response and makes it favorable for the progression of infection. ApoMVs generated during HIV infection were able to modulate the dendritic cells response *via* binding to the CD44 receptor, resulting in decrease cytokine production from dendritic cells and inhibition of their ability to prime T cells or natural killer cells (70). Second, ApoEVs could directly aid viral propagation by mediating the transfer of infectious virions to neighboring cells. Chikungunya virus was shown to induce apoptosis and the formation of ApoBDs in infected HeLa cells and blocking ApoBD formation by targeting the apoptotic cell disassembly process pharmacologically limited infection spreading to neighboring cells (71). Thus, although the formation of ApoEVs by infected cells could be beneficial for the host by facilitating the antigen presentation process, certain viruses may hijack ApoEVs to aid viral propagation.

## Conclusion

Overall, there is compelling evidence to support the importance of ApoEVs in immune modulation, and ApoEVs can play a significant role across many aspects of immunity and disease settings. Therefore, ApoEVs are more than just debris or by-products of apoptosis and should be considered as a key mechanism for apoptotic cells to communicate with surrounding cells. The ability of ApoEVs to either activate or dampen immune responses demonstrates the fine balance between the beneficial effects of ApoEV generation and the potentially damaging implications. However, as highlighted in this review, there are marked discrepancies in the characterization and isolation of ApoEVs, making it difficult to accurately define their functions. To progress the field, it is critical to identify suitable criteria to distinguish different subtypes of ApoEVs and develop better experimental systems to modulate ApoEV formation under physiologically relevant conditions.

## Author Contributions

All authors listed have made a substantial, direct, and intellectual contribution to the work and approved it for publication.

## Conflict of Interest Statement

The authors declare that the research was conducted in the absence of any commercial or financial relationships that could be construed as a potential conflict of interest.

## References

[1] Akers JC Gonda D Kim R Carter BS Chen CC. Biogenesis of extracellular vesicles (EV): exosomes, microvesicles, retrovirus-like vesicles, and apoptotic bodies. J Neurooncol (2013) 113(1):1–11. 10.1007/s11060-013-1084-8 23456661 PMC5533094

[2] Robbins PD Morelli AE. Regulation of immune responses by extracellular vesicles. Nat Rev Immunol (2014) 14(3):195–208. 10.1038/nri3622 24566916 PMC4350779

[3] Buzas EI Gyorgy B Nagy G Falus A Gay S. Emerging role of extracellular vesicles in inflammatory diseases. Nat Rev Rheumatol (2014) 10(6):356–64. 10.1038/nrrheum.2014.19 24535546

[4] Thery C Ostrowski M Segura E. Membrane vesicles as conveyors of immune responses. Nat Rev Immunol (2009) 9(8):581–93. 10.1038/nri2567 19498381

[5] Meckes DG Jr Raab-Traub N. Microvesicles and viral infection. J Virol (2011) 85(24):12844–54. 10.1128/JVI.05853-11 21976651 PMC3233125

[6] Bretz NP Ridinger J Rupp AK Rimbach K Keller S Rupp C Body fluid exosomes promote secretion of inflammatory cytokines in monocytic cells via toll-like receptor signaling. J Biol Chem (2013) 288(51):36691–702. 10.1074/jbc.M113.512806 24225954 PMC3868779

[7] Hawari FI Rouhani FN Cui X Yu ZX Buckley C Kaler M Release of full-length 55-kDa TNF receptor 1 in exosome-like vesicles: a mechanism for generation of soluble cytokine receptors. Proc Natl Acad Sci U S A (2004) 101(5):1297–302. 10.1073/pnas.0307981100 14745008 PMC337047

[8] Muller L Mitsuhashi M Simms P Gooding WE Whiteside TL. Tumor-derived exosomes regulate expression of immune function-related genes in human T cell subsets. Sci Rep (2016) 6:20254. 10.1038/srep20254 26842680 PMC4740743

[9] Qazi KR Gehrmann U Domange Jordo E Karlsson MC Gabrielsson S. Antigen-loaded exosomes alone induce Th1-type memory through a B-cell-dependent mechanism. Blood (2009) 113(12):2673–83. 10.1182/blood-2008-04-153536 19176319

[10] Raposo G Nijman HW Stoorvogel W Liejendekker R Harding CV Melief CJ B lymphocytes secrete antigen-presenting vesicles. J Exp Med (1996) 183(3):1161–72. 10.1084/jem.183.3.1161 8642258 PMC2192324

[11] Sprent J Direct stimulation of naive T cells by antigen-presenting cell vesicles. Blood Cells Mol Dis (2005) 35(1):17–20. 10.1016/j.bcmd.2005.04.004 15932799

[12] Giri PK Schorey JS. Exosomes derived from *M. bovis* BCG infected macrophages activate antigen-specific CD4+ and CD8+ T cells in vitro and in vivo. PLoS One (2008) 3(6):e2461. 10.1371/journal.pone.0002461 18560543 PMC2413420

[13] MacKenzie A Wilson HL Kiss-Toth E Dower SK North RA Surprenant A. Rapid secretion of interleukin-1beta by microvesicle shedding. Immunity (2001) 15(5):825–35. 10.1016/S1074-7613(01)00229-1 11728343

[14] Diehl P Fricke A Sander L Stamm J Bassler N Htun N Microparticles: major transport vehicles for distinct microRNAs in circulation. Cardiovasc Res (2012) 93(4):633–44. 10.1093/cvr/cvs007 22258631 PMC3291092

[15] Atkin-Smith GK Tixeira R Paone S Mathivanan S Collins C Liem M A novel mechanism of generating extracellular vesicles during apoptosis via a beads-on-a-string membrane structure. Nat Commun (2015) 6:7439. 10.1038/ncomms8439 26074490 PMC4490561

[16] Poon IKH Chiu Y-H Armstrong AJ Kinchen JM I Juncadella J Bayliss DA Unexpected link between an antibiotic, pannexin channels and apoptosis. Nature (2014) 507(7492):329–34. 10.1038/nature13147 24646995 PMC4078991

[17] Kerr JF Wyllie AH Currie AR. Apoptosis: a basic biological phenomenon with wide-ranging implications in tissue kinetics. Br J Cancer (1972) 26(4):239–57. 10.1038/bjc.1972.33 4561027 PMC2008650

[18] Ainola M Porola P Takakubo Y Przybyla B Kouri VP Tolvanen TA Activation of plasmacytoid dendritic cells by apoptotic particles – mechanism for the loss of immunologic tolerance in Sjogren’s syndrome. Clin Exp Immunol (2017) 191(3):301–310. 10.1111/cei.13077 29105068 PMC5801512

[19] Schiller M Parcina M Heyder P Foermer S Ostrop J Leo A Induction of type I IFN is a physiological immune reaction to apoptotic cell-derived membrane microparticles. J Immunol (2012) 189(4):1747–56. 10.4049/jimmunol.1100631 22786771

[20] Ferguson TA Herndon J Elzey B Griffith TS Schoenberger S Green DR. Uptake of apoptotic antigen-coupled cells by lymphoid dendritic cells and cross-priming of CD8(+) T cells produce active immune unresponsiveness. J Immunol (2002) 168(11):5589–95. 10.4049/jimmunol.168.11.5589 12023355

[21] Hochreiter-Hufford A Ravichandran KS. Clearing the dead: apoptotic cell sensing, recognition, engulfment, and digestion. Cold Spring Harb Perspect Biol (2013) 5(1):a008748. 10.1101/cshperspect.a008748 23284042 PMC3579390

[22] Poon IK Lucas CD Rossi AG Ravichandran KS. Apoptotic cell clearance: basic biology and therapeutic potential. Nat Rev Immunol (2014) 14(3):166–80. 10.1038/nri3607 24481336 PMC4040260

[23] Zitvogel L Kepp O Kroemer G Decoding cell death signals in inflammation and immunity. Cell (2010) 140(6):798–804. 10.1016/j.cell.2010.02.015 20303871

[24] Atkin-Smith GK Poon IK Disassembly of the dying: mechanisms and functions. Trends Cell Biol (2016) 27(2):151–62. 10.1016/j.tcb.2016.08.011 27647018

[25] Thery C Boussac M Veron P Ricciardi-Castagnoli P Raposo G Garin J Proteomic analysis of dendritic cell-derived exosomes: a secreted subcellular compartment distinct from apoptotic vesicles. J Immunol (2001) 166(12):7309–18. 10.4049/jimmunol.166.12.7309 11390481

[26] Tucher C Bode K Schiller P Classen L Birr C Souto-Carneiro MM Extracellular vesicle subtypes released from activated or apoptotic T-lymphocytes carry a specific and stimulus-dependent protein Cargo. Front Immunol (2018) 9:534. 10.3389/fimmu.2018.00534 29599781 PMC5862858

[27] Tixeira R Caruso S Paone S Baxter AA Atkin-Smith GK Hulett MD Defining the morphologic features and products of cell disassembly during apoptosis. Apoptosis (2017) 22(3):475–7. 10.1007/s10495-017-1345-7 28102458

[28] Lynch C Panagopoulou M Gregory CD. Extracellular vesicles arising from apoptotic cells in tumors: roles in cancer pathogenesis and potential clinical applications. Front Immunol (2017) 8:1174. 10.3389/fimmu.2017.01174 29018443 PMC5614926

[29] Wickman G Julian L Olson MF. How apoptotic cells aid in the removal of their own cold dead bodies. Cell Death Differ (2012) 19(5):735–42. 10.1038/cdd.2012.25 22421963 PMC3321633

[30] Nagata S Hanayama R Kawane K. Autoimmunity and the clearance of dead cells. Cell (2010) 140(5):619–30. 10.1016/j.cell.2010.02.014 20211132

[31] Baumann I Kolowos W Voll RE Manger B Gaipl U Neuhuber WL Impaired uptake of apoptotic cells into tingible body macrophages in germinal centers of patients with systemic lupus erythematosus. Arthritis Rheum (2002) 46(1):191–201. 10.1002/1529-0131(200201)46:1<191::AID-ART10027>3.0.CO;2-K 11817590

[32] Gaipl US Munoz LE Grossmayer G Lauber K Franz S Sarter K Clearance deficiency and systemic lupus erythematosus (SLE). J Autoimmun (2007) 28(2–3):114–21. 10.1016/j.jaut.2007.02.005 17368845

[33] Witasp E Uthaisang W Elenstrom-Magnusson C Hanayama R Tanaka M Nagata S Bridge over troubled water: milk fat globule epidermal growth factor 8 promotes human monocyte-derived macrophage clearance of non-blebbing phosphatidylserine-positive target cells. Cell Death Differ (2007) 14(5):1063–5. 10.1038/sj.cdd.4402096 17256011

[34] Moss DK Betin VM Malesinski SD Lane JD. A novel role for microtubules in apoptotic chromatin dynamics and cellular fragmentation. J Cell Sci (2006) 119(Pt 11):2362–74. 10.1242/jcs.02959 16723742 PMC1592606

[35] Casares N Pequignot MO Tesniere A Ghiringhelli F Roux S Chaput N Caspase-dependent immunogenicity of doxorubicin-induced tumor cell death. J Exp Med (2005) 202(12):1691–701. 10.1084/jem.20050915 16365148 PMC2212968

[36] Fransen JH Hilbrands LB Ruben J Stoffels M Adema GJ van der Vlag J Mouse dendritic cells matured by ingestion of apoptotic blebs induce T cells to produce interleukin-17. Arthritis Rheum (2009) 60(8):2304–13. 10.1002/art.24719 19644874

[37] Truman LA Ford CA Pasikowska M Pound JD Wilkinson SJ I Dumitriu E CX3CL1/fractalkine is released from apoptotic lymphocytes to stimulate macrophage chemotaxis. Blood (2008) 112(13):5026–36. 10.1182/blood-2008-06-162404 18799722

[38] Lauber K Bohn E Krober SM Xiao YJ Blumenthal SG Lindemann RK Apoptotic cells induce migration of phagocytes via caspase-3-mediated release of a lipid attraction signal. Cell (2003) 113(6):717–30. 10.1016/S0092-8674(03)00422-7 12809603

[39] Segundo C Medina F Rodriguez C Martinez-Palencia R Leyva-Cobian F Brieva JA. Surface molecule loss and bleb formation by human germinal center B cells undergoing apoptosis: role of apoptotic blebs in monocyte chemotaxis. Blood (1999) 94(3):1012–20. 10419893

[40] Torr EE Gardner DH Thomas L Goodall DM Bielemeier A Willetts R Apoptotic cell-derived ICAM-3 promotes both macrophage chemoattraction to and tethering of apoptotic cells. Cell Death Differ (2012) 19(4):671–9. 10.1038/cdd.2011.167 22117198 PMC3307987

[41] Eguchi A Mulya A Lazic M Radhakrishnan D Berk MP Povero D Microparticles release by adipocytes act as “find-me” signals to promote macrophage migration. PLoS One (2015) 10(4):e0123110 10.1371/journal.pone.0123110 25849214 PMC4388837

[42] Berda-Haddad Y Robert S Salers P Zekraoui L Farnarier C Dinarello CA Sterile inflammation of endothelial cell-derived apoptotic bodies is mediated by interleukin-1alpha. Proc Natl Acad Sci U S A (2011) 108(51):20684–9. 10.1073/pnas.1116848108 22143786 PMC3251090

[43] Distler JH Huber LC Hueber AJ Reich CF III Gay S Distler O The release of microparticles by apoptotic cells and their effects on macrophages. Apoptosis (2005) 10(4):731–41. 10.1007/s10495-005-2941-5 16133865

[44] Niessen A Heyder P Krienke S Blank N Tykocinski LO Lorenz HM Apoptotic-cell-derived membrane microparticles and IFN-alpha induce an inflammatory immune response. J Cell Sci (2015) 128(14):2443–53. 10.1242/jcs.162735 26034070

[45] Ravichandran KS Lorenz U. Engulfment of apoptotic cells: signals for a good meal. Nat Rev Immunol (2007) 7(12):964–74. 10.1038/nri2214 18037898

[46] Jiang L Tixeira R Caruso S Atkin-Smith GK Baxter AA Paone S Monitoring the progression of cell death and the disassembly of dying cells by flow cytometry. Nat Protoc (2016) 11(4):655–63. 10.1038/nprot.2016.028 26938116

[47] Fadok VA Voelker DR Campbell PA Cohen JJ Bratton DL Henson PM. Exposure of phosphatidylserine on the surface of apoptotic lymphocytes triggers specific recognition and removal by macrophages. J Immunol (1992) 148(7):2207–16. 1545126

[48] Verhoven B Schlegel RA Williamson P. Mechanisms of phosphatidylserine exposure, a phagocyte recognition signal, on apoptotic T lymphocytes. J Exp Med (1995) 182(5):1597–601. 10.1084/jem.182.5.1597 7595231 PMC2192221

[49] Fadok VA Warner ML Bratton DL Henson PM CD36 is required for phagocytosis of apoptotic cells by human macrophages that use either a phosphatidylserine receptor or the vitronectin receptor (alpha v beta 3). J Immunol (1998) 161(11):6250–7. 9834113

[50] Bilyy RO Shkandina T Tomin A Munoz LE Franz S Antonyuk V Macrophages discriminate glycosylation patterns of apoptotic cell-derived microparticles. J Biol Chem (2012) 287(1):496–503. 10.1074/jbc.M111.273144 22074924 PMC3249103

[51] Black LV Saunderson SC Coutinho FP Muhsin-Sharafaldine MR Damani TT Dunn AC The CD169 sialoadhesin molecule mediates cytotoxic T-cell responses to tumour apoptotic vesicles. Immunol Cell Biol (2016) 94(5):430–8. 10.1038/icb.2015.111 26647968

[52] Reich CF III Pisetsky DS. The content of DNA and RNA in microparticles released by Jurkat and HL-60 cells undergoing in vitro apoptosis. Exp Cell Res (2009) 315(5):760–8. 10.1016/j.yexcr.2008.12.014 19146850

[53] Schiller M Bekeredjian-Ding I Heyder P Blank N Ho AD Lorenz HM. Autoantigens are translocated into small apoptotic bodies during early stages of apoptosis. Cell Death Differ (2008) 15(1):183–91. 10.1038/sj.cdd.4402239 17932498

[54] Zirngibl M Furnrohr BG Janko C Munoz LE Voll RE Gregory CD Loading of nuclear autoantigens prototypically recognized by systemic lupus erythematosus sera into late apoptotic vesicles requires intact microtubules and myosin light chain kinase activity. Clin Exp Immunol (2015) 179(1):39–49. 10.1111/cei.12342 24673456 PMC4260895

[55] DeSantis CE Lin CC Mariotto AB Siegel RL Stein KD Kramer JL Cancer treatment and survivorship statistics, 2014. CA Cancer J Clin (2014) 64(4):252–71. 10.3322/caac.21235 24890451

[56] Farinacci M Weber S Kaufmann SH The recombinant tuberculosis vaccine rBCG DeltaureC:hly(+) induces apoptotic vesicles for improved priming of CD4(+) and CD8(+) T cells. Vaccine (2012) 30(52):7608–14. 10.1016/j.vaccine.2012.10.031 23088886

[57] Schaible UE Winau F Sieling PA Fischer K Collins HL Hagens K Apoptosis facilitates antigen presentation to T lymphocytes through MHC-I and CD1 in tuberculosis. Nat Med (2003) 9(8):1039–46. 10.1038/nm906 12872166

[58] Winau F Weber S Sad S de Diego J Hoops SL Breiden B Apoptotic vesicles crossprime CD8 T cells and protect against tuberculosis. Immunity (2006) 24(1):105–17. 10.1016/j.immuni.2005.12.001 16413927

[59] Dieude M Bell C Turgeon J Beillevaire D Pomerleau L Yang B The 20S proteasome core, active within apoptotic exosome-like vesicles, induces autoantibody production and accelerates rejection. Sci Transl Med (2015) 7(318):318ra200. 10.1126/scitranslmed.aac9816 26676607

[60] Braciale TJ Morrison LA Sweetser MT Sambrook J Gething MJ Braciale VL. Antigen presentation pathways to class I and class II MHC-restricted T lymphocytes. Immunol Rev (1987) 98:95–114. 10.1111/j.1600-065X.1987.tb00521.x 2443444

[61] Muhsin-Sharafaldine MR Saunderson SC Dunn AC Faed JM Kleffmann T McLellan AD. Procoagulant and immunogenic properties of melanoma exosomes, microvesicles and apoptotic vesicles. Oncotarget (2016) 7(35):56279–94. 10.18632/oncotarget.10783 27462921 PMC5302914

[62] Joffre OP Segura E Savina A Amigorena S. Cross-presentation by dendritic cells. Nat Rev Immunol (2012) 12(8):557–69. 10.1038/nri3254 22790179

[63] Fehr EM Spoerl S Heyder P Herrmann M Bekeredjian-Ding I Blank N Apoptotic-cell-derived membrane vesicles induce an alternative maturation of human dendritic cells which is disturbed in SLE. J Autoimmun (2013) 40:86–95. 10.1016/j.jaut.2012.08.003 23031801

[64] Sisirak V Sally B D’Agati V Martinez-Ortiz W Ozcakar ZB David J Digestion of chromatin in apoptotic cell microparticles prevents autoimmunity. Cell (2016) 166(1):88–101. 10.1016/j.cell.2016.05.034 27293190 PMC5030815

[65] Munoz LE Janko C Schulze C Schorn C Sarter K Schett G Autoimmunity and chronic inflammation – two clearance-related steps in the etiopathogenesis of SLE. Autoimmun Rev (2010) 10(1):38–42. 10.1016/j.autrev.2010.08.015 20817127

[66] Muhsin-Sharafaldine MR Kennedy BR Saunderson SC Buchanan CR Dunn AC Faed JM Procoagulant properties of tumor apoptotic vesicles. Biochim Biophys Acta (2016) 7(35):56279–94. 10.1016/j.bbagen.2016.11.020 PMC5302914 27462921

[67] Garg AD Krysko DV Verfaillie T Kaczmarek A Ferreira GB Marysael T A novel pathway combining calreticulin exposure and ATP secretion in immunogenic cancer cell death. EMBO J (2012) 31(5):1062–79. 10.1038/emboj.2011.497 22252128 PMC3298003

[68] Gardai SJ McPhillips KA Frasch SC Janssen WJ Starefeldt A Murphy-Ullrich JE Cell-surface calreticulin initiates clearance of viable or apoptotic cells through trans-activation of LRP on the phagocyte. Cell (2005) 123(2):321–34. 10.1016/j.cell.2005.08.032 16239148

[69] Schiller M Heyder P Ziegler S Niessen A Classen L Lauffer A During apoptosis HMGB1 is translocated into apoptotic cell-derived membranous vesicles. Autoimmunity (2013) 46(5):342–6. 10.3109/08916934.2012.750302 23194089

[70] Frleta D Ochoa CE Kramer HB Khan SA Stacey AR Borrow P HIV-1 infection-induced apoptotic microparticles inhibit human DCs via CD44. J Clin Invest (2012) 122(12):4685–97. 10.1172/JCI64439 23160198 PMC3533550

[71] Krejbich-Trotot P Denizot M Hoarau JJ Jaffar-Bandjee MC Das T Gasque P. Chikungunya virus mobilizes the apoptotic machinery to invade host cell defenses. FASEB J (2011) 25(1):314–25. 10.1096/fj.10-164178 20881210

